# Relative Localization in Wireless Sensor Networks for Measurement of Electric Fields under HVDC Transmission Lines

**DOI:** 10.3390/s150203540

**Published:** 2015-02-04

**Authors:** Yong Cui, Qiusheng Wang, Haiwen Yuan, Xiao Song, Xuemin Hu, Luxing Zhao

**Affiliations:** 1 School of Automation Science and Electrical Engineering, Beihang University, Beijing 100191, China; E-Mails: wangqiusheng@buaa.edu.cn (Q.W.); yhw@buaa.edu.cn (H.Y.); songxiao@buaa.edu.cn (X.S.); 18811435317@126.com (X.H.); 2 High Voltage Department, China Electrical Power Research Institute, Beijing 100192, China; E-Mail: zhaolx@epri.sgcc.com.cn

**Keywords:** wireless sensor networks, electric field measurement, relative localization, Received Signal Strength Indicator-based (RSSI-based)

## Abstract

In the wireless sensor networks (WSNs) for electric field measurement system under the High-Voltage Direct Current (HVDC) transmission lines, it is necessary to obtain the electric field distribution with multiple sensors. The location information of each sensor is essential to the correct analysis of measurement results. Compared with the existing approach which gathers the location information by manually labelling sensors during deployment, the automatic localization can reduce the workload and improve the measurement efficiency. A novel and practical range-free localization algorithm for the localization of one-dimensional linear topology wireless networks in the electric field measurement system is presented. The algorithm utilizes unknown nodes' neighbor lists based on the Received Signal Strength Indicator (RSSI) values to determine the relative locations of nodes. The algorithm is able to handle the exceptional situation of the output permutation which can effectively improve the accuracy of localization. The performance of this algorithm under real circumstances has been evaluated through several experiments with different numbers of nodes and different node deployments in the China State Grid HVDC test base. Results show that the proposed algorithm achieves an accuracy of over 96% under different conditions.

## 1. Introduction

High Voltage Direct Current (HVDC) transmission systems are more favorable than the conventional High Voltage Alternating Current (HVAC) systems because of their economical, technical, and environmental advantages for long distance and bulk power transmission. To date, a ±800 kV HVDC transmission system has been in commercial operation and a ±1000 kV transmission system is being studied on a research basis in China [1]. The electrical environment under HVDC transmission lines is characterized by several different electrical parameters. These parameters are the electric field, the ion current density, the space charge density, the radio interference, the audible noise and the magnetic field [2–4]. The electric field under transmission lines is one of the important parameters for evaluation of the electromagnetic environment of concern to people. Electric field measurement systems based on Wireless Sensor Networks (WSNs) have been applied extensively in the electrical power system on account of their convenience. During the process of electric field measurement, it is necessary to obtain the electric field distribution at different locations under the transmission lines, therefore, the location information of each sensor node is crucial to the analysis of the measurement results. The main focus of the research presented in this paper will be how to obtain the node location information.

To measure the electric field strength at different positions under transmission lines, electric field sensors are arrayed along the direction perpendicular to the transmission lines. The wireless node is connected with the each electric field sensor through twisted-pair braid shielded cable. The wireless nodes' primary function is to acquire and process the output analog signals of the sensor and send the digital electric field data to the remote computer. The key requirement to interpret the received electric field data is to determine the locations of the electric field sensors. The construction of the wireless nodes can be reduced to a one-dimensional linear topology where nodes have a spatial ordering. This spatial characteristic indicates the relative locations for nodes and can facilitate the establishment of the corresponding relationship between the wireless node and electric field sensor. The relative location relationship among the wireless nodes can be described as a node location permutation. A relative localization method based on Received Signal Strength Indicator (RSSI) is elaborated to obtain the correct node location permutation to get the ordering of the wireless nodes. Compared with the existing approach which gathers the location information by manually labelling sensors during deployment, automatic localization can benefit the deployment in two aspects. Firstly, in the case of a large number of nodes, the relative localization method would be more efficient and less error prone. Secondly, the relative localization method can easily re-establish the ordering in situations where a failed node is replaced or a new node is added to the current network.

The developed localization techniques can be divided into two categories: range-based and range-free [5]. Range-based localization estimates distance or angle between the unknown nodes and the anchor node, while range-free localization exploits radio connectivity to confirm proximity or exploits the sensing capabilities of each sensor. Range-based localization should estimate distance using different methods such as Time of Arrival (TOA) [6], Time Difference of Arrival (TDOA) [7], Angle of Arrival (AOA) [8], and RSSI-based ones [9–13], and then use the trilateration location algorithm, triangulation location algorithm or maximum likelihood estimation method to calculate the location of the node [14,15]. All four distance measurement methods, except for RSSI-based methods, have superior measuring precision, hence range-based localization can accomplish accurate location.

The RSSI-based localization methods can be divided into two kinds: range-based methods and range-free methods. Range-based methods assume that nodes can estimate the distance, which needs extra hardware and thus is not appropriate for large-scale outdoor sensor networks. Range-free methods localize nodes based on simple sensing features, such as wireless connectivity, anchor proximity, or localization events detection [16,17]. Among these, connectivity-based solutions feature a low cost, but at the expense of localization accuracy. Radio Frequency (RF) signals are vulnerable to environmental impact which will lead to the instability of RSSI. The environment under high voltage transmission lines is extremely complex. Electromagnetic interference, topography, vegetation, and many other factors may all have an impact on the RSSI. This will cause a large error when estimating the distance through the direct use of RSSI. Although RSSI is not considered as the best choice for distance estimation in the situation, it does provide some useful distance related information which can be used. The experimental result in the paper confirms that the radio signal strength weakens approximately monotonically with the physical distance and the RSSI could provide potential information about which neighbor node is closer and which is farther. Thus, this paper focuses on the research of range-free methods to obtain the nodes relative location information and accordingly realize the nodes sorting.

In [10], a range-free localization beyond connectivity is presented to capture the relative distance between 1-hop neighboring nodes from their neighborhood orderings that serve as unique high-dimensional location signatures for nodes in the network. The signatures are subsequently utilized to estimate the distance between nodes. In [11], one-dimensional localization algorithm based on RSSI is proposed. The algorithm utilizes the ratios of signal strengths, instead of signal strength, to determine the locations of the unknown nodes. The algorithm only needs to establish the data set for the ratio of the signal strengths once. In [12,13], the proposed algorithms assume that closer nodes receive higher RSSI. In [12], the presented algorithm can only be used in the case of the known coordinates of the database, and the wireless sensor network topology is a linear structure or a mesh structure. In [13], the algorithm that does not require the anchor node is proposed to accomplish the relative localization.

Based on the aforementioned points for network deployment, a novel and practical RSSI-based localization algorithm for WSN which assumes that closer nodes receive higher RSSI is proposed for the implementation of relative localization. The algorithm is able to correct the exceptional situation of nodes in the output location permutation to improve the accuracy of the localization. As our localization algorithm can correct RSSI-based relative localization, it is named as “Correct RSSI-Based Relative for Localization”, or “CRBR-Localization” for short.

This paper is organized as follows: in Section 2, the motivation for the CRBR-Localization algorithm is described with empirical data and comparisons with existing methods. In Section 3, the CRBR-Localization algorithm is proposed and elaborated. In Section 4, based on the experimental results, the effectiveness of the algorithm is evaluated in four different deployments. Finally, a conclusion section ends this paper.

## 2. Motivation for the Correct RSSI-Based Relative-Localization (CRBR-Localization) Algorithm

### 2.1. Empirical Data as Motivation

**Figure 1.**
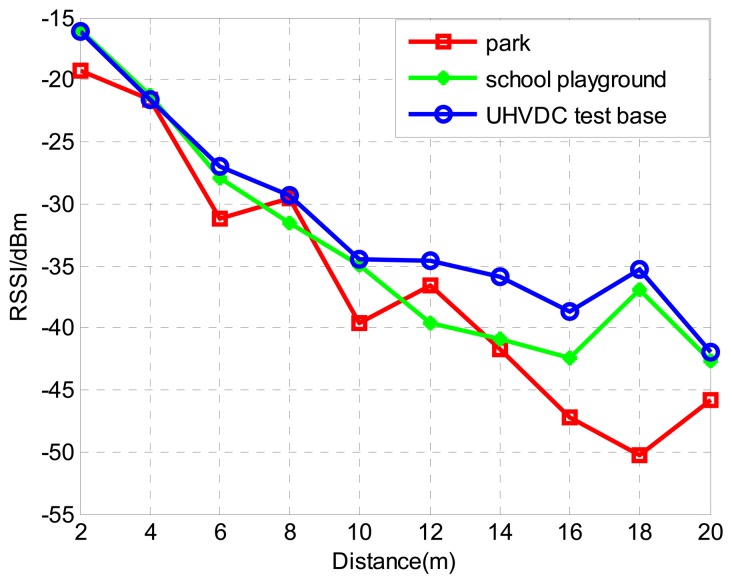
The relationship of Received Signal Strength Indicator (RSSI) and physical distance in three types of environments.

(1) Received Signal Strength Indicator (RSSI) and physical distance
  Theoretically, RSSI monotonically varies with the physical distance between the sender node and the receiver node. To evaluate the relationship of RSSI and physical distance, outdoor experiments were conducted in three types of environment: a park lawn, a school playground and the China National Grid HVDC test base. In the experiments, 10 sender nodes were arranged at different distances from the receiver node. All the nodes are located in a straight line and the interval of the sender node is 2 m. Each sender node broadcasted 19-bytes test data packets with 18 dB·m sending power, and the receiver node records the RSSI upon receiving the packet. The test is conducted 100 times and the average value of RSSI of each node from different distance is calculated. The experimental results are shown in Figure 1.
  From Figure 1, the use of RSSI for distance estimation is not reasonable since the same RSSI values could correspond to different distances. However, for each individual curve the RSSI values mostly decreased monotonically with increasing distance. There are some abnormal and exceptional situations in the curve which can be attributed to many factors, such as the RF transmission parameters, antenna issues, random noise, propagation path loss and node-level sensing discrepancy. Therefore, because of these factors, the direct use of RSSI values to calculate the physical distance of nodes will result in inaccurate results.
(2) Received Signal Strength Indicator (RSSI) ordering and node location permutation
  In a linear topology WSN, an ordered node location permutation can be formed by the nodes' physical distance. For any sender node, say *Si*, the sort order of the node location permutation, say *S*, is obtained by the sender node *Si* and the receiver node communication distance from near and far. Another permutation, say *R*, can be obtained by ordering the RSSI value from sender node *Si* to the receiver node in descending order and we define this sorted permutation as RSSI ordering. Ideally, if the RSSI decreases monotonically with the increasing distance, *S* and *R* should be identical. But due to some unexpected factors existing in the process of signal propagation, two permutations *S* and *R* could not be consistent.

**Figure 2.**
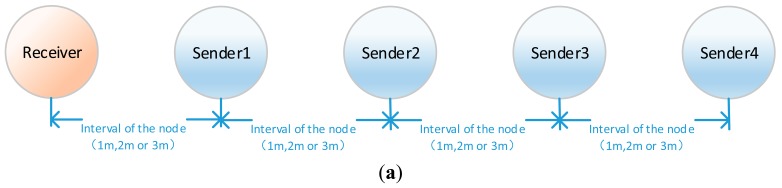

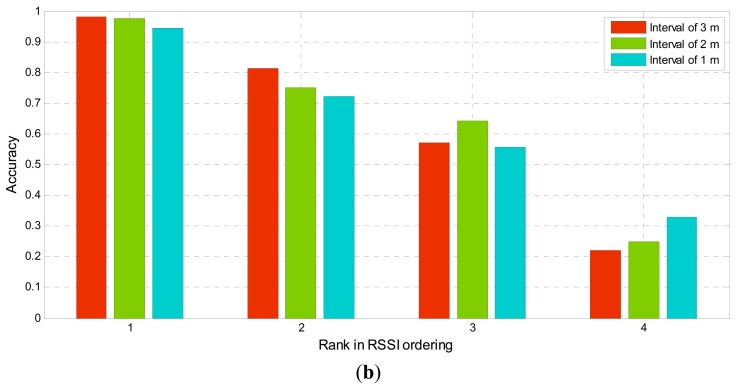
The correspondence between the Received Signal Strength Indicator (RSSI) ordering and node location permutation; (**a**) Layout of the network; (**b**) Experimental results.

As can be seen from Figure 1, the direct use of RSSI to determine the distance between the sender and receiver is not reliable. When sender nodes are far away from the receiver node, it is less accurate to infer their distance by RSSI values. Actually, this is reasonable when the process of signal propagation is considered. Signals spread during propagation and become weaker and weaker. The same noise level can influence weaker signals more easily. As a result, the RSSI becomes less reliable. It is obvious that at different distances from the receiver, the nearest node observes the best signal. In other words, the influence caused by a disturbance will be reduced as the distance between the sender and receiver decreases. This assertion has been verified in the following experiment. In the experiment, four sender nodes are arranged at different distances from the receiver node. All the nodes are in a straight line. The interval between each sender node is set to 1 m, 2 m, and 3 m, respectively. The distance between receiver and the first sender node (Sender 1) is correspondingly set to 1 m, 2 m, and 3 m. The layout of the network is shown in Figure 2a. Each sender node broadcasts 19-bytes test data packets with 18 dB m sending power, and the receiver node records the RSSI upon receiving the packet. The experiment is conducted 100 times at different intervals and the average value of RSSI of each node from different distance is calculated. The experimental result is shown in Figure 2b.

Through the experiment results, the RSSI ordering can be checked as to whether it reveals the true distance relationship among the senders. In Figure 2, the vertical ordinate indicates the accuracy of rank in the RSSI ordering compared with the corresponding rank in node location permutation. The accuracy of the nearest sender node (rank 1 in the node list) is the highest and over 94% in the case of different intervals. This percentage decreases with the increase of rank. The accuracy of the 4th nearest node (rank 4 in the node list) is lower than 32%. Although the percentage decreases with the increase of the interval, the nearest node (rank 1) still obtains a better signal than other nodes. Therefore, the assertion that the nearest node observes the best signal among nodes in different distance from the receiver is verified in the experiment and can be employed to realize node sorting.

### 2.2. Existing Methods as Motivation

The experimental results show that the nearest node usually observes the largest RSSI. Some relative localization methods employing this principle have been proposed [12,13]. The Kcd location algorithm [12] obtains the positon of unknown nodes through the relative localization between the unknown node and the anchor node. The output node permutation can be obtained through the RSSI ordering of unknown nodes. Once the location of an unknown node is known, the node can be upgraded to an anchor node and help to locate the neighboring unknown nodes. This progress is repeated until the localization of as many unknown nodes as possible has been accomplished. However, the Kcd location algorithm has two limitations: (1) it is only suitable for the case where the deployment of the sensor network has a grid or linear topology and where the interval between any two neighboring nodes is regular rather than arbitrary; (2) the Kcd location algorithm upgrades the unknown nodes which have localized their position to an anchor node status, but once an unknown node has failed to localize its positon, the subsequent unknown nodes will all fail to localize their positions.

In [13], a spatial relative localization method without the anchors has been proposed. The method consists of two parts: the first part uses a specific method to find candidate orderings, while the second part verifies the consistency of each candidate ordering and outputs the final results. For each node, it broadcasts the localization command to obtain RSSI and ID from its neighbors. All the neighbors are sorted in descending order according to their observed RSSI. The sorted list is the node neighbor list. The first part of the method is to find the location permutation of nodes with the neighbor list. It iteratively derives the node ordering and starts with each node because there is no information about the initial node. During the iteration, the method deletes the completed one from the node's neighbor list, and prepares for the next iteration until the output of node permutation. If the process cannot proceed, then the method outputs a null. The second part of the method is to verify the consistency of all the reversion of candidate permutations coming from the first part.

Compared with the Kcd location algorithm, the spatial ordering method in [13] can judge whether the output is correct. However, it cannot handle the exception of output node permutation. In practice, three kinds of results will appear when using this method to locate nodes. The first one is all the candidate permutations are wrong, then the method outputs a null; the second result is multiple candidate permutations are simultaneously consistent with the conditions, then the method outputs a null; the third result is only one permutation is consistent with the conditions and the method outputs the right permutation. The spatial ordering method also has several limitations: (1) it does not know the initial node, so it has to iteratively start with every node and derive all the nodes; (2) in the three kinds of results, only the third one can output the right permutation and the others all output a null; (3) it can only judge the correctness of the output permutation, but it cannot handle the exception of the output permutation.

## 3. Correct RSSI-Based Relative-Localization (CRBR-Localization) Algorithm

With respect to the limitations of the localization methods described in [10–13], certain improvements have been carried out and the CRBR-Localization algorithm based on the localization method in [12,13] is achieved. Compared with the localization methods in the literature [12,13], the CRBR-Localization algorithm described in the paper is more applicable in the relative localization of wireless nodes in the electric field measurement environment.

**Figure 3.**
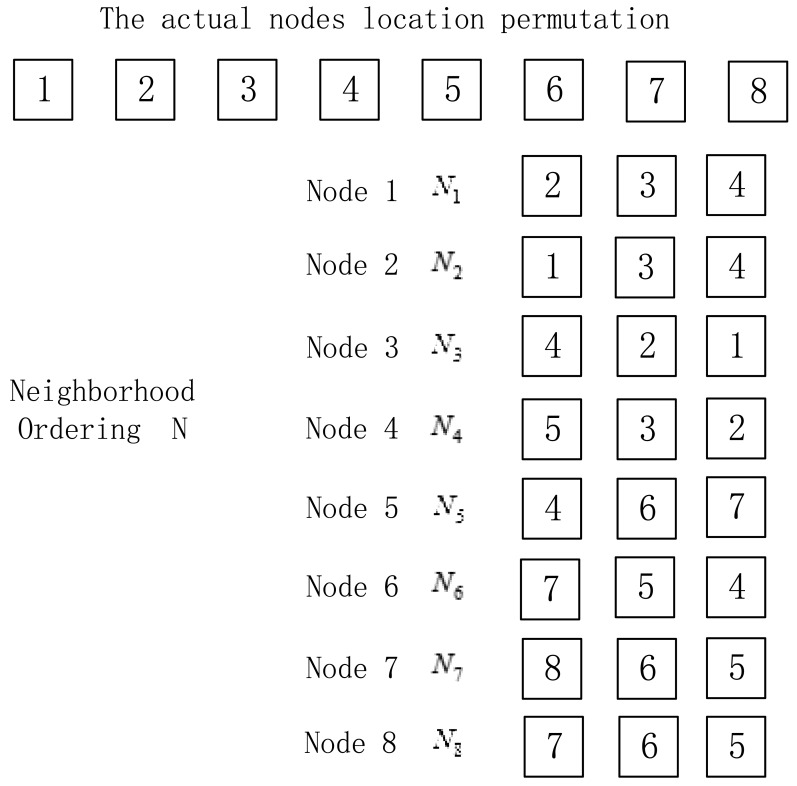
Neighbor lists.

In the localization method of the paper, the nodes that can communicate with node *i* are arranged in descending order according to the return RSSI values, thus obtaining the neighbor list of the node *i*. We select the first three nodes in the neighbor list during the localization, as shown in Figure 3.

In Figure 3, the nodes are arranged in 1–2–3–4–5–6–7–8, which is the actual nodes location permutation. *N_i_* is the neighbor list consisting of three nodes with largest return RSSI values in descending order among nodes communicating with node *i*, while *N* is the set of neighbor list *N_i_* of all nodes.

Before locating the nodes, it is necessary to mark the initial node *A* of the unknown nodes to be sorted. Taking Figure 3 for example, *S* is the output location permutation after the algorithm, and the initial node *S* (1) is node 1.We select the first node (node 2) of the neighbor list of *S* (1) as *S* (2) and delete *S* (1) in the neighbor list of all nodes; select the first node (node 3) of the neighbor list of *S* (2) as *S* (3) and delete *S* (2) in the neighbor list of all nodes. The above process is repeated until the neighbor list of the last node of the ordering is empty, and the output nodes location permutation is *S*. Now we have the first solution and process summarized in Algorithm 1.



**Algorithm 1** Deriving the permutation with initial node
**Input**: all nodes' neighborhood *N*; the initial node *A*.**Output**: node location permutation *S*.1.*S* (1) = *A*; *i* = *2;*2.**while**
*N_s_*_(_*_i_*_−1)_ is not empty **do**3. *S* (*i*) = the first element of *N_s_*_(_*_i_*_−1)_4. delete *S* (*i* − 1) in the *N*5. *i* = *i* + 16.**end while**7.output the location permutation *S*


### 3.1. Exception Handling

**Figure 4.**
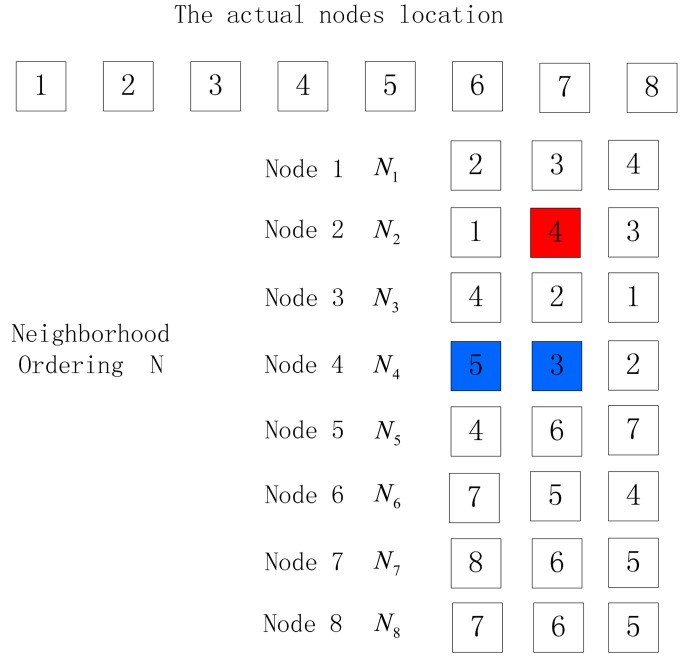
Exceptional condition 1.

**Figure 5.**
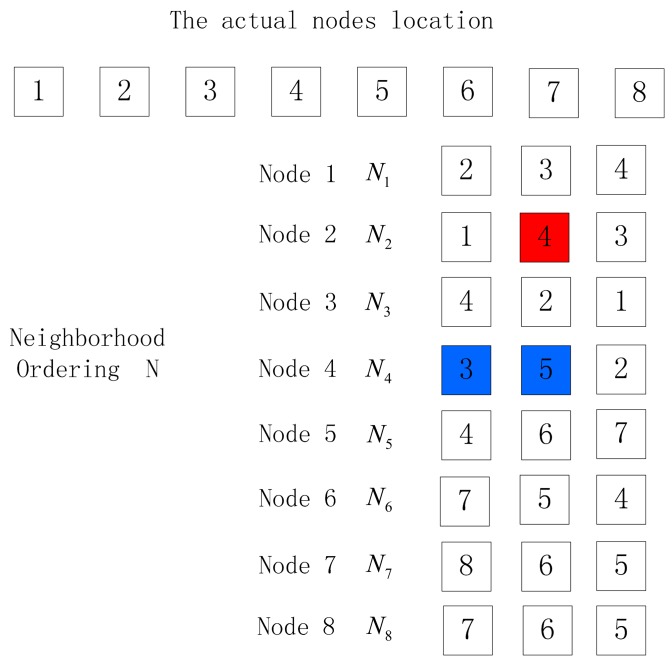
Exceptional condition 2.

**Figure 6.**
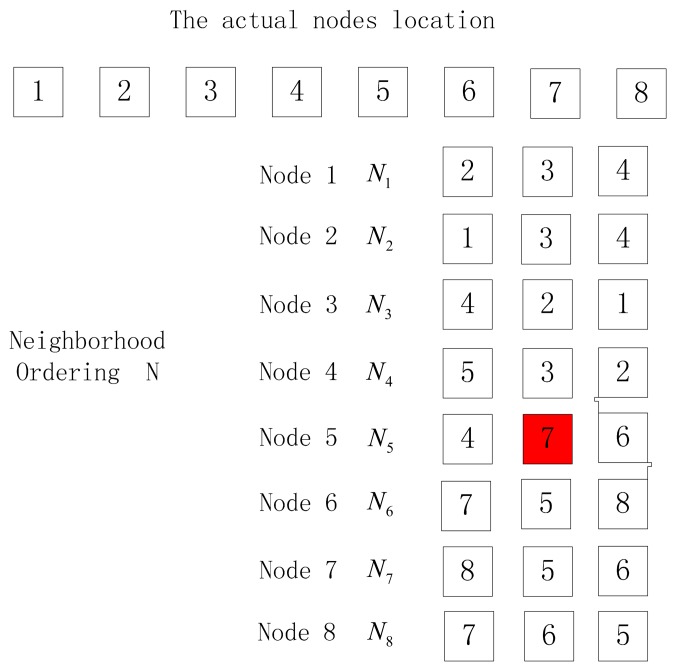
Exceptional condition 3.

**Figure 7.**
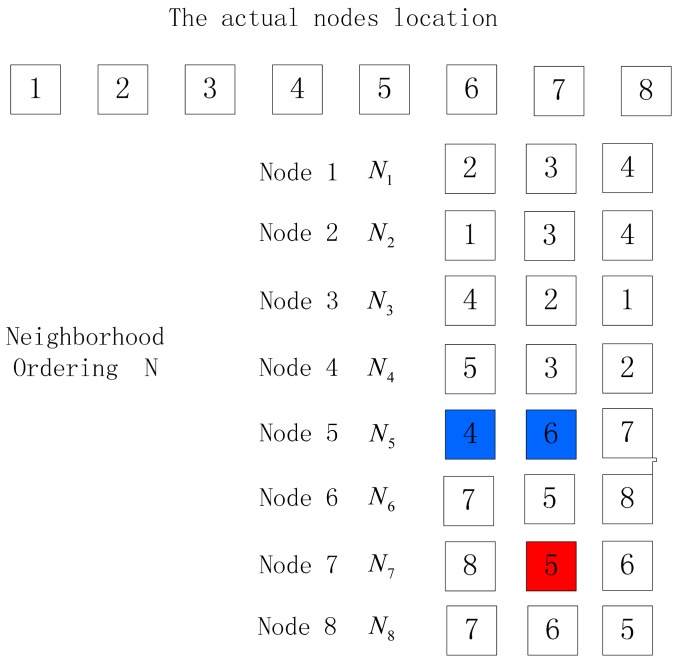
Exceptional condition 4.

**Figure 8.**
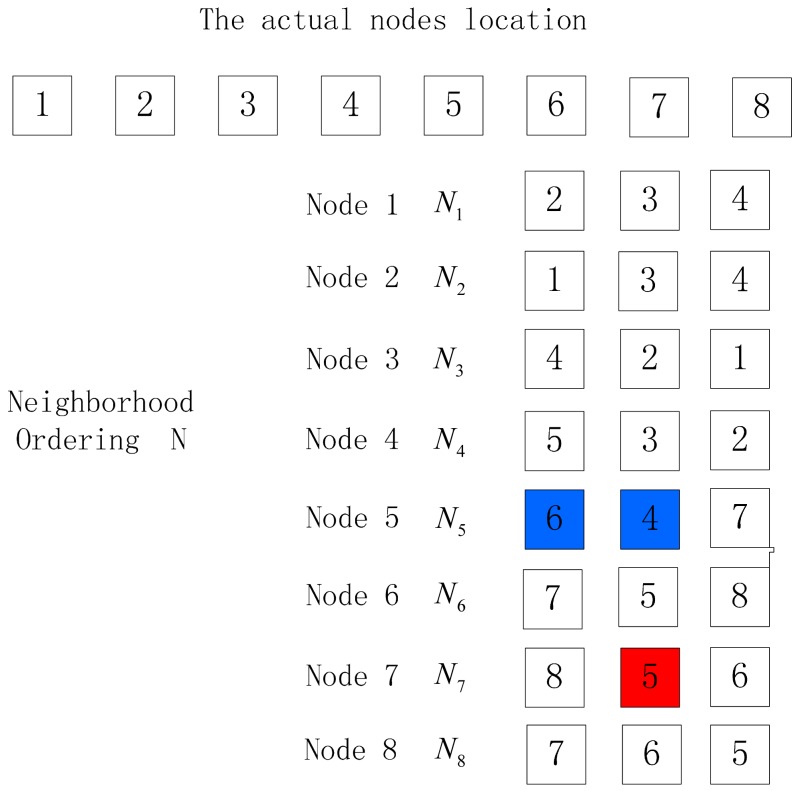
Exceptional condition 5.

From Figure 2, when the space between nodes is 1 m, over 94% of the RSSI orderings can identify the nearest node correctly, but there are still 6% of RSSI orderings failing to do so. Supposing that the neighbor node with largest RSSI value in the neighbor list of node *i* is not the nearest one to the receiver node during the location, then an inconsistent location permutation will be output. Since there is a small possibility that the above-mentioned anomalous condition may occur, we only analyze the situation where there is only one exceptional node, and the method is not applicable to the situation where several nodes are in an anomalous condition. When there is only one exceptional node among the unknown nodes to be located, two possible results arise: one is that the number of nodes in the output location permutation is less than the actual node number and another one is that the number of nodes in the output location permutation is equal to the actual node number:

(1) The number of nodes in the output location permutation is less than the actual node number.
  When there are many nodes to be sorted, there are often two things occurring when the neighbor node with the largest RSSI value is not the one nearest to the receiver node, as shown in Figures 4 and 5. In these figures, the red nodes refer to faulty neighbor nodes while the blue ones are correct neighbor nodes, but the different location of the blue nodes in the neighbor list will cause different output location permutations. In Figure 4, the output location permutation according to Algorithm 1 is 1–2–4–5–6–7–8, where the node number is one less than the actual number, while in Figure 5, the output location permutation according to Algorithm 1 is 1–2–4–3, where the node number is four less than the actual number. In these two cases, the correct location permutation fails to be output due to a certain exceptional node, so a method is desired to correct the abovementioned conditions, thus obtaining the correct output location permutation:
  (a) For the case where the number of nodes in the output location permutation is different from the actual node number by one, the following method can be employed to handle it: choose the node failing to appear in the location permutation, and check whether its first two neighbor nodes adjoin in the ordering; if so, insert the missing node between its two neighbor nodes to get a new ordering, or the sorting fails. Verify the new ordering repeatedly to determine whether it is correct, or the sorting fails. As is shown in Figure 4, Node 3 is missing in the output location permutation, and then check whether the first two nodes in the neighbor list of Node 3 adjoins in the output ordering. The first two nodes in the neighbor list of Node 3 are Node 4 and Node 2, which adjoin in the ordering 1–2–4–5–6–7–8, and then insert Node 3 between Node 4 and Node 2 to get a new ordering 1–2–3–4–5–6–7–8. Select the last node (Node 8) of the new ordering as the initial node and make reverse deduction according to Algorithm 1. If the output ordering is 8–7–6–5–4–3–2–1, it is verified to be successful and output the correct location permutation 1–2–3–4–5–6–7–8. The method to handle such an exception is as shown in Algorithm 2:

**Algorithm 2** Exception handling algorithm
**Input**: all nodes' neighborhood *N*; error location permutation *S*'.**Output**: node location permutation *S*.1.Find the missing node *M*;2.**If** the first two elements in *N_M_* are neighborhood in the error ordering *S*' **then**3.put the missing node *M* in the *S*' between the two elements in *N_M_*;4.**else**5.**break**;6.*S* (1) = *S*' (*n*), use Alogorithm1 to derive ordering *S*7.**If**
*S* is the reversion of *S*' **then**8.*S* = *S*';9.**else**10.*S* = null;11.**end if** 7. output the location permutation *S*
  (b) For the case where the difference between the number of nodes in the output location permutation and the actual node number is more than one, the following method can be employed to handle it: select the neighbor list of the last but one node in the permutation, and delete the last node of the output location permutation in its neighbor list; if its neighbor list is empty, the output is an empty set and the sorting fails; if there still is a node in its neighbor list, select this node as the next one to continue sorting. When the difference between the node number of the location permutation and the actual node number is more than one, it means more than one node are exceptional, and the sorting fails. When the difference between the node number of the location permutation and the actual node number is one, we handle it according to algorithm 2. Taking Figure 5 for example, the difference between the node number of location permutation and the actual node number is more than one and the location permutation is 1–2–4–3. Select the neighbor list *N*_4_ of the last but one node (Node 4) in the ordering and delete Node 3 from *N*_4_. Then the first neighbor node in *N*_4_ is Node 5 and make deduction according to algorithm 1 to get the order 1–2–4–5–6–7–8. Since the difference between the number of nodes in the location permutation and the actual node number is one, Algorithm 2 can be employed to handle. It can be seen that the location permutation is the same as the one in Figure 4, so Algorithm 2 can be employed to get the correct node location ordering 1–2–3–4–5–6–7–8. The detailed process is shown in Algorithm 3 to handle such an abnormity:

**Algorithm 3** Exception handling algorithm
**Input**: all nodes' neighborhood N; error location permutation *S*'.**Output**: node location permutation *S*.1.*k* = the number of *S*'; *n* = the actual node number2.delete the last element of *S*' in the *N_s_*_' (_*_k_*_−1)_3.**If**
*N_s_*_' (_*_k_*_−1)_ is null **then**4. break;5.**else**6. *S*' (*k*) = the first element of *N_s_*_' (_*_k_*_−1)_; delete *S*' (*k* − 1) in *N*7.**end if**8.**while**
*N_s_*_' (_*_k_*_)_ is not empty **do**9. *S*'(*k* + 1) = the first element of *N_s_*_' (_*_k_*_−1)_10. delete *S'* (*k*) in *N*11. *k* = *k +* 112.**end while**13.*k*' = the number of the new ordering *S*'14.**If**
*k*' = = *n*
**then**15. *S* (1) = *S*' (*n*), use Algorithm 1 to derive the ordering *S*16.   **If**
*S* is the reversion of *S*' **then**17.     *S* = *S*'18.   **else**19.     *S* = null20.   **end if**21.**else if**
*n* − *k*' = = 1 **then**22. turn to Algorithm *2*23.**else**24. *S* = null25.**end if**
(2) The number of nodes in the output location permutation is equal to the actual node number. There are often the following three things occurring when the neighbor node with the largest RSSI value measured is not the one nearest to the receiving node, as is shown in Figure 6, 7 and 8.

In Figure 6, the actual order of nodes is 1–2–3–4–5–6–7–8, but the exceptional neighbor node of Node 5 causes the ordering output according to algorithm 1 to be 1–2–3–4–5–7–8–6. When in such a case, the reverse ordering of the output location permutation, *S* (*n*) *S* (*n* − 1) *…*, *S* (1), can be verified to determine whether the output location permutation is correct, that is to take *S* (*n*) as the initial node and make ordering deduction according to algorithm 1 to get the location permutation, which is called the reverse verification permutation S1. If the output reverse verification permutation is identical to the reverse ordering of permutation S, the sorting is correct, and if not, the sorting is faulty. In Figure 6, permutation *S* is 1–2–3–4–5–7–8–6, and the reverse verification ordering deducted with Node 6 as the initial node is 6–7–8–5–4–3–2–1, which is not consistent with the expected reverse verification permutation, 6–8–7–5–4–3–2–1, so the node sorting is faulty.

In Figure 7, the actual order of nodes is 1–2–3–4–5–6–7–8, and although the neighbor node of Node 7 is exceptional, the ordering output according to Algorithm 1 is 1–2–3–4–5–6–7–8. However, when taking Node 8 as the start node, the reverse verification ordering output according to Algorithm 1 is 8–7–5–4–3–2–1, which is not consistent with the expected reverse verification ordering, 8–7–6–5–4–3–2–1.

In Figure 8, the actual order of nodes is consistent with that in Figure 7, and its ordering permutation according to Algorithm 1 is still 1–2–3–4–5–6–7–8, but its reverse verification permutation according to Algorithm 1 is 8–7–5–6, which is not consistent with the expected reverse verification ordering, 8–7–6–5–4–3–2–1.

The three cases mentioned above can be handled according to the following algorithm. When permutation *S* is deducted according to Algorithm 1,whether the output location ordering is correct can be determined by comparing whether the reverse verification permutation *S*1 is consistent with the reverse ordering of permutation *S*. If the node number of permutation *S*1 is identical to that of permutation *S*, and it is the reverse ordering of permutation *S*, the sorting is correct; if the difference between the node numbers of permutation *S*1 and permutation *S* is one, it can be corrected according to algorithm 2 using *S*1 as input; if the difference between the node numbers of permutation *S*1 and permutation *S* is more than one, it can be corrected according to Algorithm 3 using *S*1 as input; if it cannot be corrected, the sorting fails. When in the three above-mentioned cases, only Figure 6 fails to output correct ordering while in the other two cases, relevant algorithms can be used to get correct node location ordering. The detailed process is shown in Algorithm 4.



**Algorithm 4** Exception handling algorithm
**Input**: all nodes' neighborhood *N*; verified permutation *S***Output**: node location permutation *S*.1.use Alogorithm1 to derive the reverse verification permutation *S*12.*k* = the number of ordering *S*1; *n* = the actual node number3.**If**
*k* = = *n*
**then**4. **If**
*S*1 is reversion of *S*
**then**5. *S* = *S*6. **else**7. *S* = null8. **end if**9.**else if**
*n* – *k* = = 1 **then**10. turn to Algorithm 2 *using S*1 *as input*11.**else**12. turn to Algorithm 3 *using S*1 *as input*13.**end if**


### 3.2. Realization of Correct RSSI-Based Relative-Localization *(*CRBR-Localization*)* Algorithm

The CRBR-Localization algorithm combines the two methods in [12,13], and applies a correction algorithm with respect to their respective shortcomings, which make it possible to handle the exception where one node fails. However, CRBR-Localization algorithm is only capable of correcting the cases in Figures 4, 5, 6, 7 and 8, and for the case where several nodes fail, the algorithm output ordering is empty. The realization of the CRBR-Localization algorithm is shown in Algorithm 5:

**Algorithm 5** CRBR-Localization Algorithm
**Input**: all nodes' neighborhood *N*; the initial node *A*; the actual node number *n*.**Output**: node location permutation *S*.1.use Algorithm 1 to derive the permutation *S*2.*k* = the number of ordering *S*3.**If**
*k* = = *n*
**then**4. turn to Algorithm 45.**else if**
*n* − *k* = = *1*6. turn to Algorithm 27.**else**8. turn to Algorithm 39.**end if**


## 4. System Implementation and Evaluation

To evaluate the CRBR-Localization algorithm, three indexes are chosen: (1) percentage of output, which is the ratio of the occurrences that the output permutation is a non-empty set after sorting to the experiment occurrences; (2) output accuracy, which is the ratio of the occurrences that the output permutation is a right sequence to the occurrences the output permutation is a nonempty set after sorting; (3) accuracy, the ratio of the occurrences that the output permutation is a right one after sorting to the experiment occurrences. The proposed three indexes can be defined as Equations (1)–(4):

(1) $$R_{po}=\frac{N_{\text{non}}}{N_{\text{all}}}$$

(2) $$R_{oa}=\frac{N_{\text{acu}}}{N_{\text{non}}}$$

(3) $$R_{\text{acu}}=\frac{N_{\text{acu}}}{N_{\text{all}}}$$

(4) $$R_{\text{acu}}=R_{po}\times R_{oa}$$

where *R_po_* is percentage of output, *R_oa_* is output accuracy, *R_acu_* is accuracy, *N_non_* is the occurrences that the output permutation is a nonempty set after sorting, *R_acu_* is the occurrences that the output permutation is a right sequence after sorting, *R_all_* is the total experiment occurrences. From the definitions of the three indexes, we can know that accuracy (*R_acu_*) equals to the arithmetic product of the percentage of output (*R_po_*) and the output accuracy (*R_oa_*). The ideal algorithm has not only a high percentage of output but also high output accuracy. Meanwhile, the index of accuracy could be a comprehensive evaluation of the algorithm's percentage of output and output accuracy.

### 4.1. Experimental System

**Figure 9.**
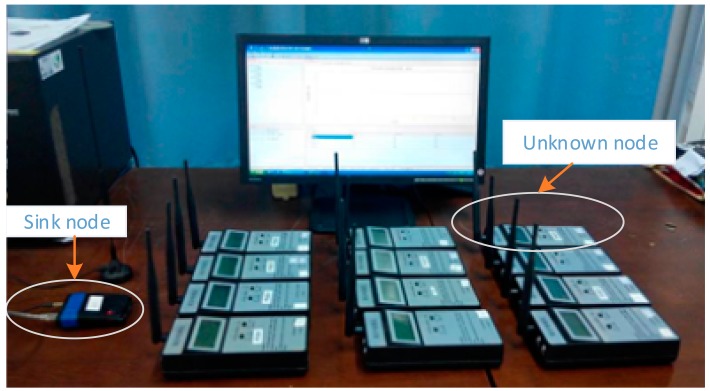
Experiment system at the High Voltage Direct Current (HVDC) test base of the China Electric Power Research Institute.

To validate and verify the proposed localization algorithm, a double circuit experimental line on the same tower was erected at the HVDC test base of China Electric Power Research Institute in Beijing. Figure 9 shows our hardware system for the localization.

**Figure 10.**
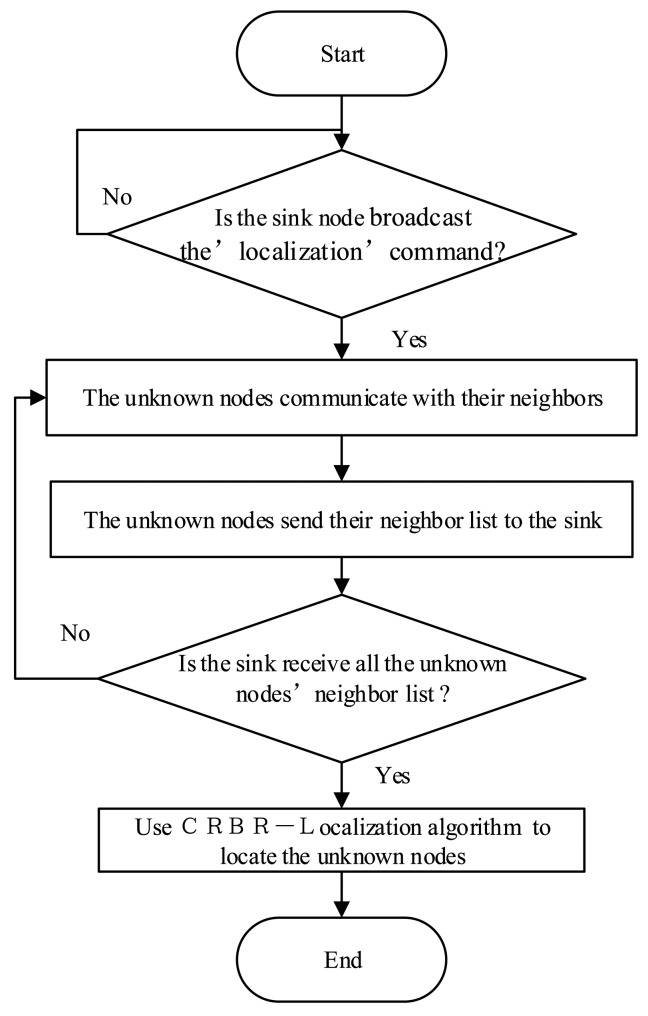
Workflow of the Correct RSSI-Based Relative-Localization (CRBR-Localization) algorithm.

There are two kinds of nodes which are wireless nodes (unknown nodes) and a sink node in the system. The sink node is connected to the computer through the USB bus. The wireless nodes which are arranged in a line perpendicular to the transmission lines are the subject of the localization. The sink node broadcasts the localization command message to the wireless nodes. Upon receiving the message, the wireless nodes will communicate with their neighbor nodes and obtain the neighbor list. In the same way, all the wireless nodes in turn get their neighbor lists and then transmit them to the sink node. As a result, the computer connected with the sink node executes the CRBR-Localization algorithm. The detailed workflow of the CRBR-Localization algorithm is shown in Figure 10. The sink node has two main functions. The first one is to broadcast the localization commands and another one is to receive all the unknown nodes' neighbor lists used for the CRBR Localization algorithm. There is a high-performance CPU and enough memory in each unknown node, so the neighbor list of each unknown node is created by the unknown node itself and then sent to the sink node. Therefore in the experimental system, only one node which is connected with the computer through the USB interface is the sink node. The sink node could be outside or at one end of the network of unknown nodes. It is convenient for the operator to always take as the sink node the one with the connected computer.

**Figure 11.**
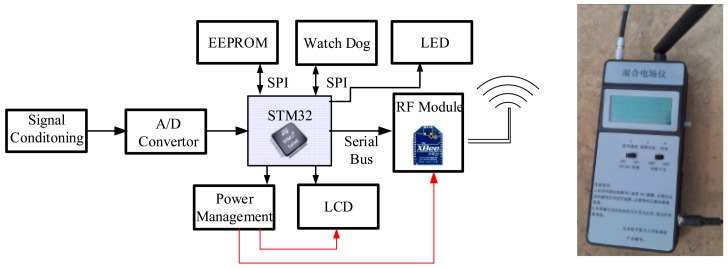
Structure and picture of a wireless node.

A block diagram of the proposed wireless node is presented in Figure 11. The wireless nodes' primary function is to acquire and process the output analog signals from the electric field sensors and send the digital data to the sink node. The core of the wireless node is an STM32F embedded microcontroller. The wireless communication system selected for the sensor node is the 2.4 GHz Xbee Pro Radio Frequency (RF) module based on the IEEE 802.15.4 protocol from Digi^TM^ (Minnetonka, MN, USA)

### 4.2. Node Number Experiments

**Figure12.**
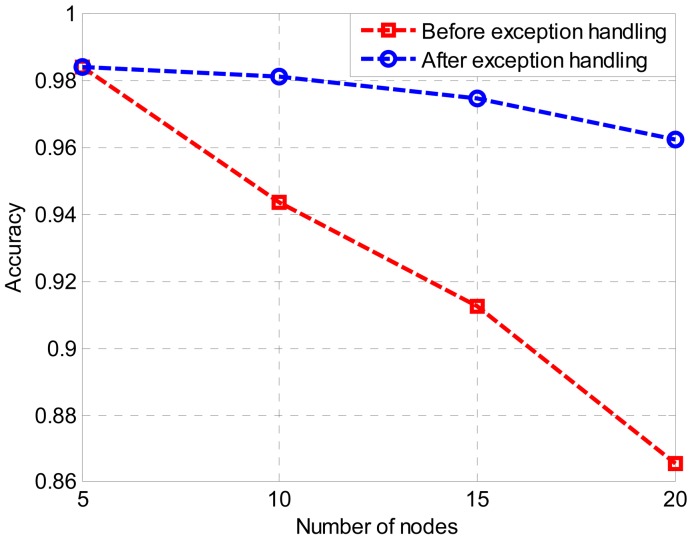
Experimental results.

In the electric field measurement environment, since the transmission line spacing is different, different numbers of sensor nodes are needed. In order to verify the validity of the algorithm, we did sorting experiments using different node numbers. In these experiments, the numbers of unknown nodes to be positioned are 5, 10, 15, and 20, respectively. The nodes are arranged in a line perpendicular to the transmission lines, and the interval between nodes is 2.5 m. The RSSI values that are returned by the unknown nodes in each sequence are sampled five times and averaged in order to use the average for sorting. Three hundred sequences are obtained with each sorting, and the sorting accuracy is counted and analyzed. The result of the experiment is shown in Figure 12.

Since the output accuracies of the experiments are all 100%, the accuracy equals the percentage of output, thus only accuracy is used as the evaluation criterion. From Figure 12 we can see that when sorting five nodes, the sorting accuracies of nodes before and after exception handling are both 98.4%, thus when the number of nodes is small, exception handling has an insignificant influence on the result. Node sorting accuracy before exception handling falls dramatically with the increase of the number of sorting nodes. When the number of sorting nodes is 10, the accuracy is 94.4%, and when the number of sorting nodes is 15, the accuracy is 91.25%. When the number of sorting nodes is 20, the accuracy is only 86.6%. Node sorting accuracy after exception handling falls slightly with the increase of the number of sorting nodes. When the number of sorting nodes is 10 the accuracy is 98.1%, and when the number is 15, the accuracy is 97.5%. When the number of sorting nodes is 20, the accuracy is still above 96.3%, so when the number of nodes in the experiment is large, the sorting accuracy can be improved markedly through exception handling by the CRBR-Localization algorithm.

### 4.3. Experiment in Four Deployments

Theoretically, when placing sensor nodes, every node should be arranged in a line perpendicular to the transmission lines. However, since there exist personal deployment errors, not all the nodes are in a straight line, so the adaptability of the CRBR-Localization algorithm to different node deployment structures needs to be verified.

In the experiment, we sequence 10 unknown wireless nodes in four different deployment structures. The RSSI values that are returned by the unknown nodes in each sorting are sampled five times and averaged for further sorting. Three hundred sequences are obtained with each structure. The results are obtained to compare with the Spatial Ordering method presented in [13] and the Kcd location algorithm presented in [12].

#### 4.3.1. Linear Homogeneous Deployment

**Figure 13.**
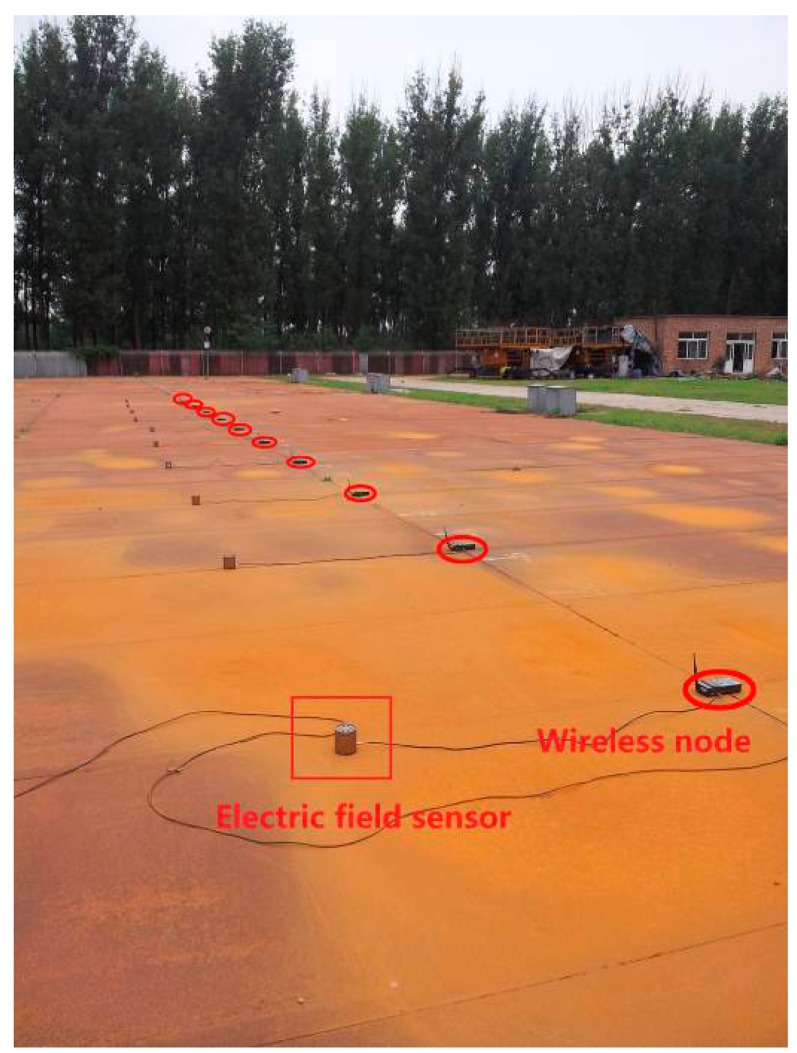
Linear homogeneous deployment structure.

**Figure 14.**
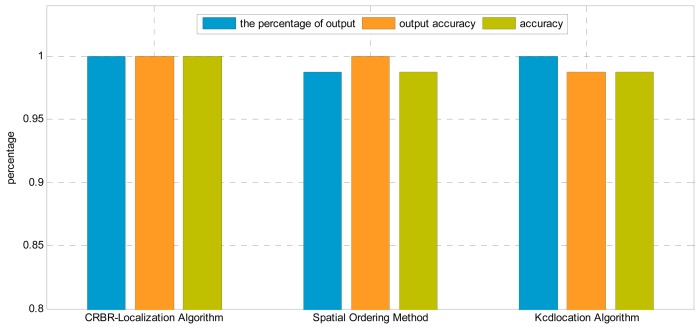
Localization results comparison by using different algorithms in the linear homogeneous deployment.

In this experiment, the interval between neighboring nodes is 3 m. The nodes are arranged in a line perpendicular to the transmission line, as shown in Figure 13. The distance between the first node and the last node is 27 m, and they can communicate normally. The result is shown in Figure 14.

From Figure 14 we can see that the percentage of output and the output accuracy of the CRBR-Localization algorithm are both 100%, higher than the values of the other two algorithms. Since the Spatial Ordering method doesn't process exceptional conditions and may have several candidate orderings, its percentage of output is lower than that of the CRBR-Localization algorithm and Kcd location algorithm. Because the Kcd location algorithm can't judge the correctness of its output sequences, although the percentage of output is 100%, the output accuracy is still lower than those of the CRBR-Localization algorithm and Spatial Ordering method. Comprehensively comparing the three algorithms, we can see that the three indexes of the CRBR-Localization algorithm are all higher than those of the other two algorithms, and it has higher localization accuracy.

#### 4.3.2. Linear Inhomogeneous Deployment

**Figure 15.**
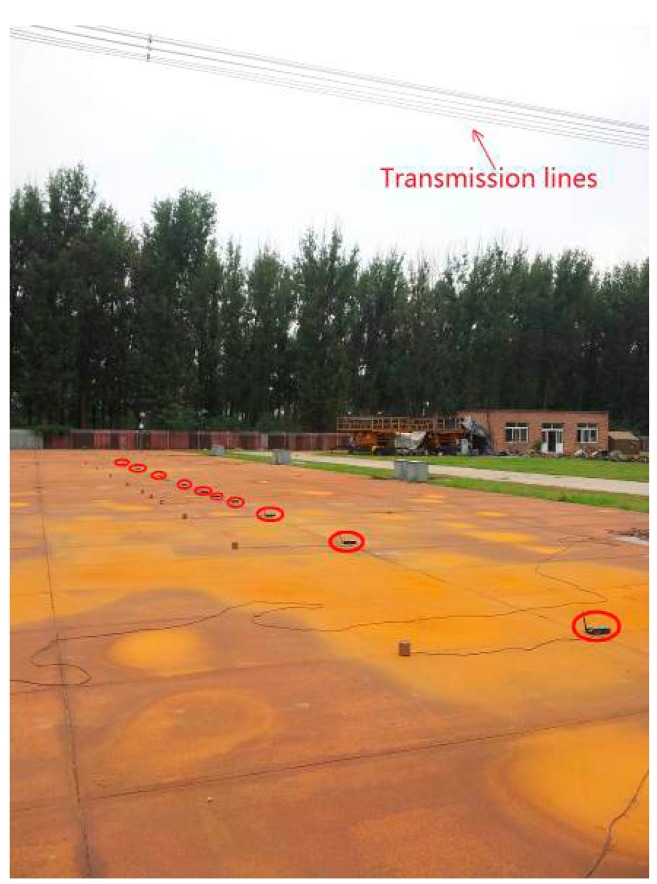
Linear inhomogeneous deployment structure.

**Figure 16.**
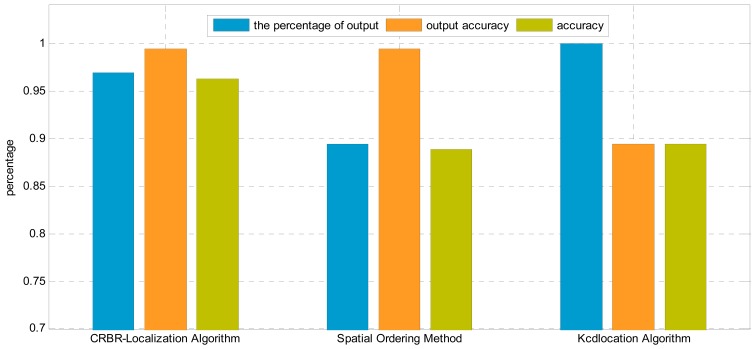
Localization results comparison by using different algorithms in the linear inhomogeneous deployment.

In this experiment, the intervals between neighboring nodes are not the same. The maximum interval is 4 m, while the minimum is 1.5 m. The nodes are arranged in a line perpendicular to the transmission lines, as shown in Figure 15. The first node and the last node can communicate normally, as shown in Figure 16.

From Figure 16, Kcd location algorithm has the highest percentage of output, and the CRBR-Localization algorithm is in the middle, while the Spatial Ordering method has the lowest, which is less than 90%. Although the Kcd location algorithm has the highest percentage of output, its accuracy and output accuracy are both lower than 90%, apparently lower than those of the CRBR-Localization algorithm.

The percentage of output of the CRBR-Localization algorithm is higher than 99%, and its accuracy is higher than 96%, both of which are higher than Kcd location algorithm and Spatial Ordering method. Under linear inhomogeneous deployment conditions, the percentage of output of CRBR-Localization algorithm and Spatial Ordering method are both lower than under a linear homogeneous alignment. This is because when the nodes are placed homogeneously, there may be a situation that some nodes are placed closely and others are placed loosely. When the nodes are close, the distance between nodes is small, so the RSSI values measured are close. Since the RSSI values of each node are close, elements like electromagnetic components, topographic relief and surface vegetation will interfere with the radio signal, which will have a greater influence on obtaining the RSSI accurately, whereas the CRBR-Localization algorithm and the Spatial Ordering method can judge the correctness of output location permutation, and when the output permutation is judged as a wrong one, then output will not be allowed. In addition to that, the CRBR-Localization algorithm can not only judge the correctness of output permutations, but also further correct the wrong permutations, so it has a higher percentage of output and accuracy than the Spatial Ordering method.

#### 4.3.3. Cross Deployment

**Figure 17.**
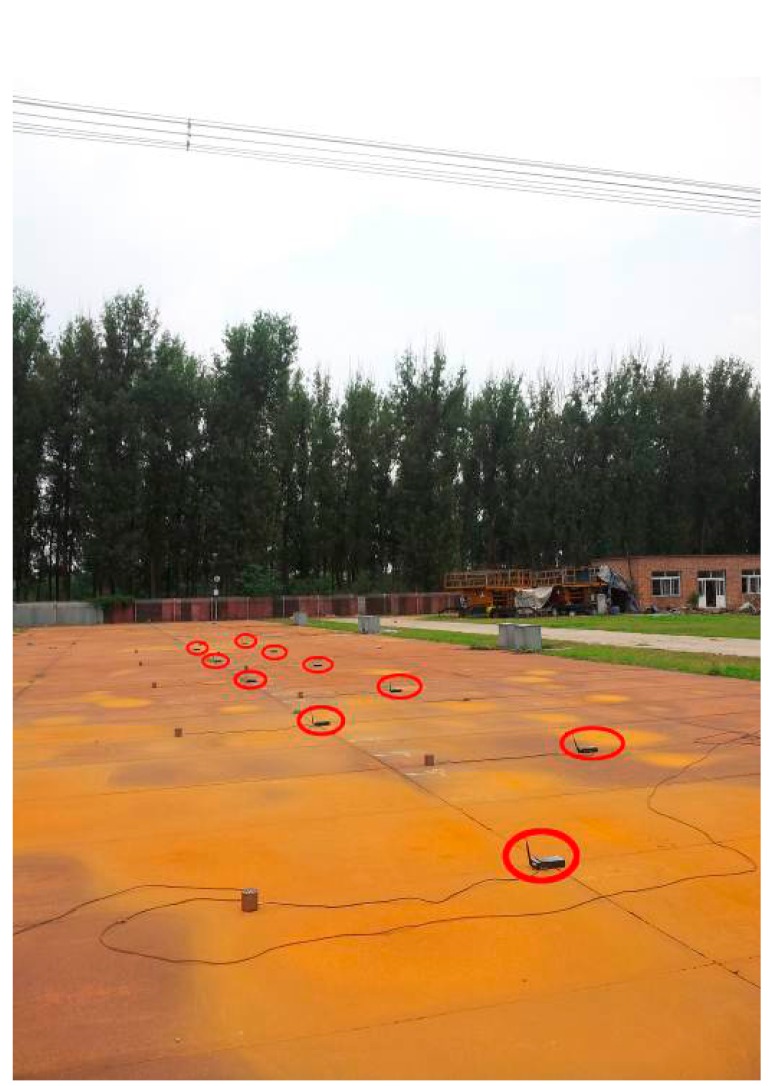
Cross deployment structure.

**Figure 18.**
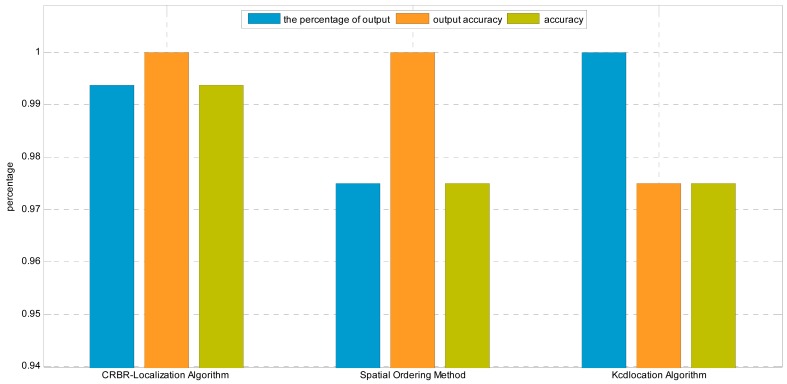
Localization results comparison by using different algorithms in the cross deployment.

In this experiment, each node is first arranged in a line with a 3 m interval. Then we make each node move 0.5 m to the left and right, respectively, as is shown in Figure 17. From Figure 18, the CRBR-Localization algorithm has an output accuracy of 100%, and has a higher accuracy than the Kcd location algorithm and Spatial Ordering method. Though the Kcd location algorithm has a high percentage of output, it can't judge the correctness of the output location permutations, so its percentage of output and accuracy are both lower than those of the CRBR-Localization algorithm. Comprehensively comparing the three algorithms, we can see that in cross deployment structure scenarios, the overall performance of the CRBR-Localization algorithm is better than that of the other two algorithms.

#### 4.3.4. Random Deployment

**Figure 19.**
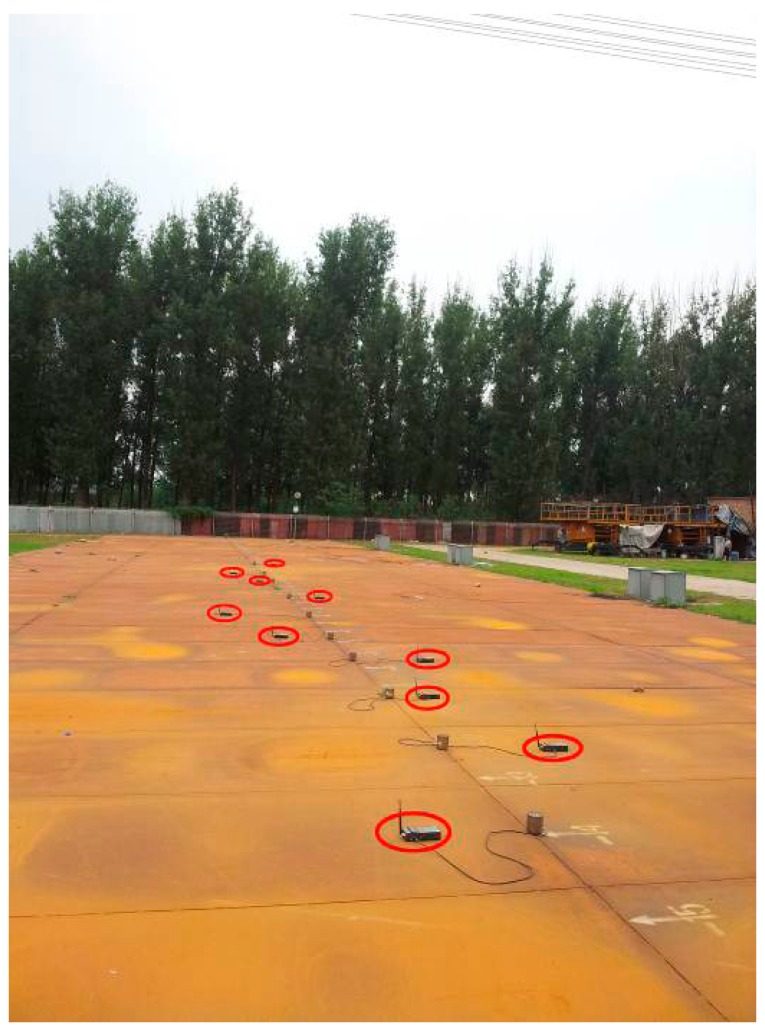
Random deployment.

In actual measurement, since there exist personal deployment errors, so not all the nodes are arranged according to the ideal structure (linear and homogeneous). Therefore, an approximate random arrangement is used to simulate the situations in an actual measurement environment. Under this deployment, the nodes are arranged randomly in a rectangle 30 m long and 1 m wide, and the minimum interval of adjacent nodes is 1.8 m, while the maximum is 3.6 m, as shown in Figure 19.

**Figure 20.**
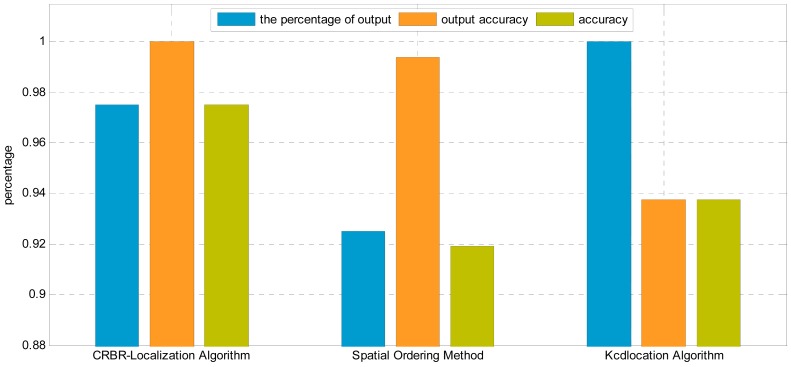
Localization results comparison by using different algorithms in the random deployment.

Under random deployment, the intervals between nodes are not the same, and not all the nodes are in the same line, but they are randomly arranged in a rectangle area. Under this circumstance, the RSSI is sensitive to the interference factors in the environment. From Figure 20, we can see that the CRBR-Localization algorithm has accuracy and percentage of output higher than 97%, and its output accuracy is 100%. Both its output accuracy and its percentage of output are higher than those of the Kcd location algorithm and Spatial Ordering method. Though the Spatial Ordering method has a higher percentage of output, its accuracy and output accuracy are both lower than 94%, which is lower than the CRBR-Localization algorithm. Comprehensively comparing the three algorithms, we can see that under random arrangement conditions, the overall performance of the CRBR-Localization algorithm is better than that of the other two algorithms. In the four different localization experiments, the accuracy and percentage of output of the CRBR-Localization algorithm are apparently higher than those of the Kcd Location algorithm and the Spatial Ordering method.

Four different deployment structures, the accuracies of the CRBR-Localization algorithm are all above 96%. Taking the three performance indexes into consideration, we can see that compared with the other two algorithms, the CRBR-Localization algorithm has a better overall performance, and has a better adaptability of different applied environments of electric field measurement.

## 5. Conclusions

In this paper, a RSSI-based algorithm is proposed for achieving the relative localization of one-dimensional linear topology wireless networks under HVDC transmission lines. Starting from the observation that the closest node always has the highest RSSI, the algorithm utilizes unknown nodes' neighbor list based on the RSSI values to determine the relative locations of nodes. A detailed interpretation has been made around the solutions of the exceptional situations for the output permutation which can effectively improve the localization accuracy. The performance of this algorithm in real circumstances has been evaluated through several experiments with different numbers of nodes and different deployments at the China State Grid HVDC test base. Results show that in the experiments with different number of nodes, the accuracy of the CRBR-Localization algorithm is above 96.2% and in the experiments with different deployments the accuracy of the CRBR-Localization algorithm, which is over 96%, is higher than that of the other two algorithms.
